# Different Dimensions of Cognitive Style in Typical and Atypical Cognition: New Evidence and a New Measurement Tool

**DOI:** 10.1371/journal.pone.0155483

**Published:** 2016-05-18

**Authors:** Andy D. Mealor, Julia Simner, Nicolas Rothen, Duncan A. Carmichael, Jamie Ward

**Affiliations:** 1 School of Psychology, University of Sussex, Falmer, Brighton, BN1 9QH, United Kingdom; 2 Department of Psychology, University of Edinburgh, 7 George Square, Edinburgh, EH8 9JZ, United Kingdom; University of Plymouth, UNITED KINGDOM

## Abstract

We developed the Sussex Cognitive Styles Questionnaire (SCSQ) to investigate visual and verbal processing preferences and incorporate global/local processing orientations and systemising into a single, comprehensive measure. In Study 1 (*N* = 1542), factor analysis revealed six reliable subscales to the final 60 item questionnaire: *Imagery Ability* (relating to the use of visual mental imagery in everyday life); *Technical/Spatial* (relating to spatial mental imagery, and numerical and technical cognition); *Language & Word Forms*; *Need for Organisation*; *Global Bias*; and *Systemising Tendency*. Thus, we replicate previous findings that visual and verbal styles are separable, and that types of imagery can be subdivided. We extend previous research by showing that spatial imagery clusters with other abstract cognitive skills, and demonstrate that global/local bias can be separated from systemising. Study 2 validated the *Technical/Spatial* and *Language & Word Forms* factors by showing that they affect performance on memory tasks. In Study 3, we validated *Imagery Ability*, *Technical/Spatial*, *Language & Word Forms*, *Global Bias*, and *Systemising Tendency* by issuing the SCSQ to a sample of synaesthetes (*N* = 121) who report atypical cognitive profiles on these subscales. Thus, the SCSQ consolidates research from traditionally disparate areas of cognitive science into a comprehensive cognitive style measure, which can be used in the general population, and special populations.

## Introduction

An individual’s cognitive style refers to his or her preferred method for acquiring and processing information and this is regarded as relatively stable over time. Cognitive styles can encompass attitudes, preferences or strategies used by individuals that influence functions such as perceiving, remembering, thinking, and problem solving [1] (see [2], for review). We aimed to develop a new questionnaire—the *Sussex Cognitive Styles Questionnaire* (SCSQ)–to investigate the interrelationships between visual and verbal cognitive styles and to incorporate recent findings on global/local processing biases and systemising. To preface our study, we describe below the different facets of cognitive styles that one might wish to capture in a comprehensive cognitive styles questionnaire. We then describe the design of our own questionnaire and the way we have validated it in different populations of typical and atypical individuals.

One traditional view of visual and verbal cognitive styles places those who prefer visual processing strategies and those who prefer verbal processing strategies as on opposite ends of a visual-verbal continuum [3,4]. That is, visualisers who prefer to use visual mental imagery to process information are placed at one end of the continuum and verbalisers, who prefer verbal techniques, are on the other. The self-report *Verbaliser-Visualiser Questionnaire* (VVQ; [4]) was originally designed to classify people along this dimension. Recent neuroimaging results support the distinction between visualisers and verbalisers. For example Kraemer, Rosenberg and Thompson-Schill [5], demonstrated that participants who score highly on verbal preference exhibit greater activation in phonological regions when presented with pictorial stimuli, and those who score highly on visual preference exhibit greater activation in visual cortex when presented with written descriptions of visual stimuli (see also [6,7]). However, research has also demonstrated independence between visual and verbal dimensions on self-report measures [8,9], and it is therefore theoretically possible for an individual to score high (or low) on both visualising and verbalising [10].

Recent research has shown visual style can be further subdivided into two relatively independent imagery factors—object imagery and spatial imagery [11,12]. Object imagery refers to the subjective quality of cognitive representations in terms of resemblance to an object’s form (encompassing, for example, size, shape, colour, brightness), whereas spatial imagery refers to the quality of representations in terms of spatial relationships between objects (encompassing, for example, location, movement, spatial transformation). Blajenkova et al. [11] developed the two-factor self-report *Object-Spatial-Imagery Questionnaire* (OSIQ) to capture dissociations between object and spatial imagery, with items such as “My images are very colourful and bright” loading on to the former factor and items such as “I was very good in 3D geometry as a student” loading on to the latter. Object imagery scores correlated with performance on a degraded pictures task (identifying a picture from partial information) and spatial imagery correlated with a performance on paper folding and mental rotation tasks, thereby demonstrating predictive validity of the measure. Blazhenkova and Kozhevnikov [13] subsequently developed the *Object-Spatial Imagery and Verbal Questionnaire* (OSIVQ) to include a third factor representing verbal style. This factor predicted performance on a task of generating sentences using a set of four nouns.

Spatial mental imagery, in particular, has been shown to correlate with another cognitive style, namely systemising [14–16]. Systemising refers to the drive to understand rules, regularities or variables that govern how a system works, or the tendency to construct systems to understand the world [17–19]. Systemising can be gauged via the self-report questionnaire called the *Systemising Quotient* (SQ; [19]). Behavioural studies have shown that systemising is correlated with the ability to mentally rotate 3D shapes [15], and other aspects of mental rotation such as stimulus encoding and same-different comparison processes [14]. Furthermore, scores on the short-form SQ [15] correlate well with the spatial imagery subscale of the OSIVQ (*r* = .61; [16]). Spatial abilities such as mental rotation, or everyday activities such as map reading or solving the Rubik’s cube, are thought to involve systemising as input features must be transformed via the application of rules in order to arrive at the correct outcome [17]. The present research considers whether systemising and spatial imagery reflect a unitary cognitive style or two separate, but related, processes.

Systemising also presupposes excellent attention to detail [20] and as such bears at least a superficial similarity to a local processing bias. When attending to a complex stimulus, such as a scene, one can focus on it holistically or focus on its constituent parts. These can be thought of as global (holistic) or local (featural or analytic) processing biases respectively. Systemising is presumably dependent on sufficient local processing for relevant task details to be attended to and then acted upon. Indeed, Billington, Baron-Cohen and Bor [21] demonstrated that scores on the *Revised Systemising Quotient* (SQ-R; [22]) correlate with local bias in the Navon [23] letters task (*r* = .45).

Global/local biases can influence visual processing more generally. For example, a global bias is positively correlated with performance on judgments of line orientation [24] and inappropriate local processing orientations can disrupt recognition of complex visual stimuli such as faces and cars [25–27]. Additionally, Bouvet, Rousset, Valdois and Donnadieu [28] found correlations between local and global processing across different modalities (e.g., a participant exhibiting a faster response time in identifying global patterns in visual stimuli was likely to exhibit a similar response time advantage for global audio stimuli, *r* = .55), and they suggest that global/local biases may be indicative of a particular cognitive style (see also [29] for a similar suggestion). Thus there is some support for the notion that global/local biases represent somewhat stable traits. The key difference between local processing and systemising is that systemising requires understanding the operations involved in a task and noting the consequences of actions [18]. A local processing bias does not in and of itself presuppose a drive to understand the relations between elements in a problem, but does suggest a predisposition towards attention to detail. Thus it is not clear whether systemising tendencies and global/local bias would be driven by the same latent factor, or whether they are largely independent. Despite this, we are aware of no published self-report measure which gauges whether a person thinks of themselves as naturally inclined towards local or global processing, which we address here. Furthermore, the relationship between systemising and local processing, on the one hand, and visual-verbal measures of cognitive style, on the other, is essentially unknown.

We sought to design a comprehensive new questionnaire measure—the SCSQ—with items designed to capture self-reported imagery, systemising and global/local bias. We aimed to replicate the finding that visual and verbal styles can be divided, and that certain types of self-reported mental imagery can be further subdivided. We also aimed to extend the scope of such measures by additionally considering systemising and global/local bias. Specifically, we aimed to determine whether systemising and global/local bias items would or would not cluster together (cf [21]), and how these variables would relate to different forms of visual imagery (cf [13]), as it was unclear a priori whether (some) systemising and global/local bias items would cluster with aspects of visual imagery.

Study 1 describes the new SCSQ, and presents factor and reliability analyses as well as the correlations between factor scores. Gender differences are also considered. Following the factor analysis and subscale establishment in Study 1, we aimed to validate the SCSQ in two further studies. Study 2 sought to validate relevant subscales of the SCSQ via correlation with performance on visual and verbal memory tasks in a subset of participants from Study 1.

Study 3 aimed to validate the SCSQ via testing on a group of unusual individuals. We asked whether the presence of certain forms of synaesthesia—which have been linked to particularly vivid imagery (e.g., [10,30])–would predict scores on SCSQ subscales. Individuals with synaesthesia have additional perceptual-like experiences (‘concurrents’) in the presence of certain materials (‘inducers’), such as experiencing colours when reading or listening to music [31], and we might therefore expect the presence of certain forms of synaesthesia to be predictive of visual, verbal, systemising and global/local processing preferences in particular ways compared to non-synaesthetes. We focus on two variants of synaesthesia. Firstly grapheme-colour synaesthesia, where letters and/or numbers result in additional colour experiences (e.g., the letter A might be crimson red; for review see [32]), and secondly sequence-space synaesthesia, where particular sequences (e.g., numbers, months, years) are consciously visualised as having spatial arrays (e.g., the months of the year might be experienced in an ellipse in front of the body, with January, say, to the right; for review see [33]). Following the results of the factor analysis in Study 1, we were able to make predictions about how synaesthetes’ cognitive styles may differ from those of non-synaesthetes, and thereby validate the SCSQ subscales. We address this in Study 3.

## Study 1

The aim of Study 1 was to design a new cognitive styles questionnaire (the SCSQ). We then sought to assess its factor structure and reliability, explore the correlations between the factors, and investigate gender differences on the obtained factors.

### Method

#### Participants

One thousand five hundred and forty-two participants took part in our study and completed the questionnaire (956 female, 586 male). Ages ranged from 16 to 90 (*M* = 27, *SD* = 13). As the questionnaire only asks for birth year, not date of birth, the ages are estimated. Participants were recruited from the student population at the University of Sussex and from volunteers at the University of Edinburgh. Sussex participants (*n* = 370) were recruited in exchange for course credit. Edinburgh participants (*n* = 1172) were recruited as part of a large-scale, centrally co-ordinated undergraduate research project. Every student registered on the 2^nd^ year of the Psychology undergraduate course at the University of Edinburgh acted as a research assistant, and each recruited 8 participants (4 male and 4 female) over 16 years of age.

#### Ethics statement

The study procedures and methods of providing consent for all three studies in the current paper were approved by the local ethics committees at the Universities of Sussex and Edinburgh. On the first page of the questionnaire, participants were informed they would be asked to consent to taking part in the study. If they wished to participate, they continued to the second page and clicked a button, agreeing to complete the measure and for their data to be used. The Sussex cohort were undergraduates aged 18 or over. The Edinburgh cohort were recruited from student and non-student populations. The ethics committee who approved the study confirmed that, as per British Psychological Society guidelines, no specific consent procedures were required for prospective participants aged 16 and over, and these participants could therefore provide consent for themselves. Thus, 16–17 year olds taking part in this study used the standard consent procedure provided to all participants.

#### Design of the questionnaire

Eighty-four items were initially included on the questionnaire (see S1 Table for the list of items). Previous questionnaires such as the OSIVQ [13] were developed on the basis that items should reflect preferences for the use of object, spatial and verbal processing in everyday life. Therefore, we aimed to select and generate items which could reasonably be said to reflect everyday events, activities or affairs for the SCSQ.

Twenty-two items were taken from the OSIVQ, 11 of which loaded on to their Object Imagery factor and 11 on to their Spatial Imagery factor. Four items were additionally taken from the ‘Habitual Use of Imagery’ subscale of the *Individual Differences Questionnaire* (IDQ; [3,9]). The IDQ measures imagery and verbal thinking habits. The Habitual Use of Imagery subscale was most relevant to our research question (i.e. processing preferences in everyday life). Twenty-four items were taken from the SQ to assess systemising [19]. Seven items reasonably related to systemising or global/local biases were additionally taken from the ‘Attention to Detail’ subscale of the *Autism Quotient* (AQ; [34]).

Twenty-seven further items were generated by us to measure how global/local processing bias, systemising, imagery, and verbal habits may be used in everyday life. In particular, we generated five items concerned with the visual appearance of facets of language (e.g., *I tend to notice if a word has the same letter repeated in its spelling*). We did this to determine whether verbal items that focus on the visual appearance of written language would form a separate factor from visual imagery items more generally. All items were measured on a five point Likert scale (from ‘strongly disagree’ to ‘strongly agree’, with the midpoint being ‘neither agree nor disagree’) in line with the measurement scale of the OSIVQ (note the original AQ and SQ are measured on four point scales). The IDQ was originally measured on a true/false response scale, but performs well when a five-point scale is used instead [9,35]. The presentation order of items was randomised, but fixed between participants (see S1 Table).

#### Procedure

Questionnaire data were collected via the online portal Bristol Online Surveys. All participants completed the SCSQ in a location of their choosing, aside from 44 Sussex participants who completed it under laboratory conditions (as part of Study 2). After providing consent to participate on the first and second web page, participants were asked to enter their demographic information (gender, birth year, level of achieved formal education, nationality). All questionnaire items were presented on the next webpage. The final page gave a brief description of grapheme-colour and sequence-space synaesthetic experiences and asked the question–“do you suspect you have synaesthesia?”. This final question was for the purposes of screening out potential synaesthetes at this stage from the initial factor structure analysis as this forms the basis of a separate study.

### Results and Discussion

Of the 1542 participants who completed the measure, 87 (5.6%) reported they thought they may experience some form of synaesthesia and these participants were excluded from further consideration. The analyses were conducted on the remaining 1455 participants (907 females, 548 males). Age data were available for 1437 of these participants (range 16–90 years; *M* = 27; *SD* = 13). Data are included in S1 File.

#### Exploratory factor analysis

Exploratory factor analysis (EFA) with weighted least squares estimation was conducted on all 84 items to determine the model that best described the data. Spearman correlations were used in the analysis due to the ordinal nature of the items. Initially, we determined the number of factors to extract using Velicer’s [36] Minimum Average Partial (MAP) method. This suggested a nine factor solution at this stage. Thus an EFA was specified with a nine factor solution, with an oblique rotation (Oblimin-Quartimin) to allow for correlations between factors. Oblimin rotations parametrise the obliqueness of the rotated factors. Quartimin rotations are a special case, where the solution is most oblique by minimising the product of the squared loadings on each factor. Items with factor loadings > .30 were considered worthy of retention. Seventeen items did not load at .30 at this stage and were removed from further analysis (Q03, Q12, Q13, Q14, Q34, Q35, Q37, Q39, Q49, Q50, Q68, Q70, Q74, Q75, Q76, Q79, Q83). Five further items were removed for cross-loading on more than one factor (Q26, Q54, Q63, Q66, Q82).

A second MAP analysis was conducted, suggesting a six factor solution to the remaining 62 items. This resulted in a clear and interpretable factor structure. Regarding this second EFA, the Kaiser-Meyer-Olkin measure of sampling adequacy suggested a factorable solution (KMO = .87). At this stage, two remaining items did not load at the .30 level on to the obtained factors and were removed from further consideration (Q30, Q80). Table 1 presents the results of the EFA. The reduced version of the questionnaire contained sixty items, which explained 32% of the total variance. See Tables A-F in S2 Table for the inter-item correlation matrices for the six obtained factors.

**Table 1:** Rotated factor matrix of factor loadings for the final sixty items of the SCSQ.

Item	Imagery Ability (F1)	Technical / Spatial (F2)	Language and Word Forms (F3)	Need for Organisation (F4)	Global bias (F5)	Systemising Tendency (F6)
I often use mental images or pictures to help me remember things.^a^	0.711					
Mental imagery helps me remember things.^b^	0.703					
My memories are mainly visual in nature.^b^	0.634					
I often enjoy the use of mental pictures to reminisce.^a^	0.623					
My mental images are very vivid and photographic.^c^	0.623					
When I think of activities I have done, I do not remember in mental pictures.^b^	-0.615					
I can close my eyes and easily picture a scene that I have experienced.^c^	0.606					
When reading fiction, I usually form a clear and detailed mental picture of a scene or room that has been described.^c^	0.514					
My mental images of different objects very much resemble the size, shape and colour of actual objects that I have seen.^c^	0.449					
When remembering a scene, I use verbal descriptions rather than mental pictures.^a^	-0.445					
When I can't find something I'm looking for, I automatically visualise the last place I saw it.^b^	0.442					
I have only vague visual impressions of scenes I have experienced.^a^	-0.425					
When I picture the route somewhere, I visualise that route as if I were walking/driving/cycling it.^b^	0.375					
When entering a familiar store to get a specific item, I can easily picture the exact location of the target item, the shelf it stands on, how it is arranged and the surrounding articles.^c^	0.372					
My mental images are more schematic than colourful and pictorial.^d^	-0.342					
When I'm planning to do a complex or difficult task, I visualise myself doing it first.^b^	0.313					
When I hear a radio announcer or a DJ I've never actually seen, I usually find myself picturing what he or she might look like.^c^	0.311					
I can easily imagine and mentally rotate three-dimensional geometric figures.^d^		0.742				
In school, I had no problems with geometry.^d^		0.641				
I find it difficult to imagine how a three-dimensional geometric figure would exactly look like when rotated.^d^		-0.639				
In maths, I am intrigued by the rules and patterns governing numbers. ^e^		0.593				
My graphic abilities would make a career in architecture relatively easy for me.^d^		0.578				
I am fascinated by numbers.^f^		0.577				
I am good in playing spatial games involving constructing from blocks and paper (e.g., Lego, Tetris, Origami).^d^		0.568				
I find it easy to grasp exactly how odds work in betting.^e^		0.512				
I have excellent abilities in technical graphics.^d^		0.494				
I can easily sketch a blueprint for a building I am familiar with.^d^		0.485				
I prefer schematic diagrams and sketches when reading a textbook instead of colourful and pictorial illustrations.^d^		0.483				
If I were buying a stereo, I would want to know about its precise technical features.^e^		0.448				
If I were buying a computer, I would want to know exact details about its hard drive capacity and processor speed.^e^		0.447				
When I read the newspaper, I am drawn to tables of information, such as football league scores or stock market indices.^e^		0.403				
I notice patterns in things all the time.^f^		0.376				
I do not enjoy games that involve a high degree of strategy.^e^		-0.376				
When thinking about an abstract concept (or building), I imagine an abstract schematic building in my mind or its blueprint rather than a specific concrete building.^d^		0.349				
I like learning new words.^b^			0.711			
When I hear a new word, I am curious to know how it is spelled.^b^			0.670			
The spelling of words does not fascinate me.^b^			-0.655			
When I read something, I always notice whether it is grammatically correct.^e^			0.558			
I tend to notice if a word has the same letter repeated in its spelling.^b^			0.438			
I enjoy learning languages.^b^			0.386			
I keep my workspace highly organised (e.g., all files/folders on the same subject are in the same colour).^b^				0.674		
If I had a collection (e.g., CDs, coins, stamps), it would be highly organised.^e^				0.609		
Order is important to me.^b^				0.563		
I like to group things together under a single label.^b^				0.539		
I find it easy to group things together under a single label.^b^				0.413		
I keep my book collection organised alphabetically.^b^				0.353		
I usually concentrate on the whole picture, rather than the small details.^f^					0.615	
I tend to focus on details in a scene rather than the whole picture.^b^					-0.598	
I can easily remember a great deal of visual details that someone else might never notice. For example, I would just automatically take some things in, like what colour is a shirt someone wears or what colour are his/her shoes.^c^					-0.558	
I tend to notice details that others do not.^f^					-0.498	
I don't usually notice small changes in a situation or a person's appearance.^f^					0.427	
I tend to omit small visual details in scenes I remember.^b^					0.346	
When I think of a face, I imagine it as a whole rather than focus on individual features.^b^					0.332	
When I am walking in the country, I am curious about how the various kinds of trees differ.^e^						0.627
I do not care to know the names of the plants I see.^e^						-0.522
I am interested in knowing the path a river takes from its source to the sea.^e^						0.505
When I look at an animal, I like to know the precise species it belongs to.^e^						0.467
I am fascinated by dates.^f^						0.421
When I think of historical events, the exact date is important to me.^b^						0.385
When I look at a tree I focus on its features such as branches and leaves rather than the whole.^b^					-0.305	0.308
**Number of items**	**17**	**17**	**6**	**6**	**8**	**6**
**Percentage explained variance**	**11**	**8**	**5**	**3**	**3**	**2**
**Ordinal alpha**	**.88**	**.89**	**.80**	**.77**	**.74**	**.73**
**Mean score (SE)**	**3.60 (.01)**	**2.92 (.02)**	**3.52 (.02)**	**3.09 (.02)**	**2.99 (.02)**	**2.63 (.02)**

Note.

^a^From IDQ [9].

^b^New item.

^c^. OSIVQ—object [13].

^d^OSIVQ—spatial [13].

^e^SQ [19].

^f^AQ—attention to detail [40].

Factor 1 contained items mostly related to the use of visual imagery in everyday life (e.g., “I often use mental images or pictures to help me remember things” from the IDQ), with some items which gauge the subjective quality of visual imagery (e.g., “My mental images are very vivid and photographic” from the OSIVQ), hence this factor was termed ‘***Imagery Ability****’*. Factor 2 contained items relating to spatial mental imagery (e.g., “I can easily imagine and mentally rotate three-dimensional geometric figures” from the OSIVQ), but also items relating to mathematics abilities (e.g., “I am fascinated by numbers” from the AQ) and items relating to technical interests (e.g., “If I were buying a computer, I would want to know exact details about its hard drive capacity and processor speed” from the SQ), thus this factor was termed ‘***Technical*/*Spatial****’*. All items on Factor 3 related to aspects of language. In particular most of these items indicate an interest in the visual appearance of written language as opposed to oral verbal ability (e.g., “When I hear a new word, I am curious to know how it is spelled”, a new item; “When I read something, I always notice whether it is grammatically correct”, from the SQ). These items clearly formed their own factor separate from visual imagery. Therefore this factor was termed ‘***Language* & *Word Forms****’*.

Factor 4 contained items relating to organisation (e.g., “If I had a collection (e.g., CDs, coins, stamps), it would be highly organised” from the SQ) and order (e.g., “Order is important to me”, a new item) and was termed ‘***Need for Organisation****’*. Negatively loaded items on Factor 5 indicate attention to detail or a local processing preference (e.g., “I tend to focus on details in a scene rather than the whole picture”, a new item), whereas positively loaded items indicate a more holistic preference (e.g., “I usually concentrate on the whole picture, rather than the small details” from the AQ), therefore this factor was termed ‘***Global Bias****’*. Finally, items on Factor 6 refer to the drive to categorise (e.g., “When I look at an animal, I like to know the precise species it belongs to” from the SQ), or interest in systems (e.g., “I am fascinated by dates” from the AQ) and was termed ‘***Systemising Tendency****’*. One item (“When I look at a tree I focus on its features such as branches and leaves rather than the whole”) cross-loaded onto two factors: Factors 5 and 6. This item better captures attention to detail or local bias and was therefore included on Global bias (Factor 5). Interestingly, as both the globally and locally phrased items loaded on to the same factor as opposed to two different factors, it seems that these preferences are at opposite ends of a spectrum in terms of self-report.

#### Reliability analysis

Next, we assessed the reliability of the obtained factors via ordinal α (see Table 1). All factors exhibited good levels of reliability (all α ≥ .73). Next we consider improvements to α with the removal of items. The reliability of *Imagery Ability* would not improve with the removal of any item. The reliability of *Technical/Spatial* would increase from .885 to .887 with the removal of Q48. The reliability of *Language & Word Forms* would increase from .798 to .804 with the removal of Q1. The reliability of *Need for Organisation* would increase from .770 to .776 with the removal of Q38. The reliability of *Global Bias* would increase from .736 to .746 with the removal of Q27. The reliability of *Systemising Tendency* would not benefit from the removal of any item. As the removal of any item would only result in minor improvement in ordinal α (≤ .01) all items were deemed worthy of retention.

Next we calculated the mean scores for each factor (negatively loading items were reverse coded prior to calculation; see Table 1 for mean factor scores). Table 2 displays the correlation coefficients between the six obtained factors. Although most factors tended to correlate the effect sizes were small (*r*s < .30) in all but one case (based on the categorisation of effect sizes by Cohen [37]). *Global Bias* and *Systemising Tendency* correlated negatively, and one might expect sufficient local processing to systemise effectively. *Systemising Tendency* correlated positively with *Technical/Spatial*, showing that spatial skills and systemising are related. *Technical/Spatial* also correlated negatively with *Global Bias*. It is not surprising that *Systemising Tendency* and *Technical/Spatial* were correlated; one might expect that sufficient local detail is needed for spatial transformation and attention to detail in systemising domains. *Imagery Ability* also correlated negatively with *Global Bias*.

**Table 2:** Correlation coefficients (Pearson’s *r*) between scores on the scales of the SCSQ.

Factor	F1	F2	F3	F4	F5
1 Imagery Ability					
2 Technical/Spatial	.10***				
3 Language & Word Forms	.16***	.05			
4 Need for Organisation	.14***	.22***	.12***		
5 Global Bias	-.31***	-.14***	-.17***	-.13***	
6 Systemising Tendency	.12***	.21***	.27***	.15***	-.23***

Note.

*** *p* < .001.

#### Gender differences

Next we assessed gender differences on SCSQ factor scores. Mean scores for females and males were calculated and compared via independent samples *t*-tests (Table 3).

**Table 3:** Mean factor scores as a function of gender.

Gender	Imagery Ability	Technical / Spatial	Language & Word Forms	Need for Organisation	Global Bias	Systemising Tendency
Male	3.55 (.02)	3.27 (.03)	3.47 (.03)	3.08 (.03)	3.02 (.02)	2.72 (.03)
Female	3.68 (.02)	2.70 (.02)	3.54 (.02)	3.10 (.02)	2.98 (.02)	2.58 (.02)
*t*(1453)	4.69***	18.26***	1.63	0.39	1.60	3.86***
Cohen’s *d*	0.25	1.00	0.08	0.03	0.09	0.20

Note.

*** *p* < .001. Standard errors appear in parentheses.

Consistent with Blazhenkova and Kozhevnikov [13], females scored higher on *Imagery Ability* (similar to their object imagery factor) and males scored higher on *Technical/Spatial* (similar to their spatial factor, with additional mathematics and technical interest items). Similar results for imagery according to gender are also presented by Campos [38]. Males also scored higher than females on Systemising Tendency, consistent with Baron-Cohen et al. [19], Ling et al. [15] and Wheelwright et al [22].

## Study 2

As the SCSQ used items pertaining to the everyday use of dimensions on the measure, the goal of Study 2 was to validate the visual and verbal subscales of the new measure by correlating mean factor scores with performance on modified versions of tasks taken from a standard memory battery, the Wechsler Memory Scale Revised (WMS-R; [39]). Specifically, we adapted the visual paired associates and verbal paired associates tasks. Both tasks included additional trials to prevent ceiling effects in the current student sample (the original versions of these tasks are used in clinical samples), and both tasks were computerised using E-Prime 2 software. We also sought to validate the measure with an additional visual recognition task using fractal images as stimuli. The fractal image recognition task has previously been used by Ward, Hovard, Jones and Rothen [40]. The task was chosen here because the visual stimuli are hard to verbalise and performance on the task tends to be overall lower and more variable than those used in the WMS-R (which was designed to measure memory impairment).

### Method

#### Participants

Forty-four participants recruited from the University of Sussex (37 female, 7 male) who had provided data for Study 1 completed these additional tasks. Ages ranged from 18 to 42 years (*M* = 21; *SD* = 4). All had normal or corrected to normal vision and had a good level of English. None indicated they experienced any form of synaesthesia. Prior to the task, participants signed a consent form, thereby providing written consent to take part in the memory tasks. Consent to complete the questionnaire was acquired in the same way as Study 1.

#### Materials and procedure

The visual paired associates task requires participants to remember specific colours associated with monochrome line drawings. The original task utilises six of these colour-shape pairs. We included four additional pairs, meaning ten pairs were used in total. Each pair consisted of two boxes, with the shape on the left hand side and a square of colour on the right hand side. The screen background colour was white. The task alternated between three learning blocks and three memory blocks with a final delayed memory block which was completed after a retention interval of approximately thirty minutes. During the learning blocks, each colour-shape pair was presented for 3 seconds, in the centre of the screen (with a 3 second inter-trial interval). Here, participants were instructed to memorise the pairings. During the memory blocks, a shape prompt appeared in the top third of the screen. All ten colours appeared in a row at the bottom third of the screen, which corresponded to keys 1 to 0 on the top number pad of the keyboard. Participants were required to pick the colour associated with the shape via the corresponding key input (this response was unspeeded), and the next prompt appeared. This continued for all ten pairs. Corrective feedback was provided onscreen (duration of 1.5 seconds). Participants completed the delayed block without an additional learning block, and no feedback was provided during this block. Presentation order of pairings was pseudorandomised between participants.

The verbal paired associates task required the participants to associate two words in memory. The original version of the task includes eight pairs of concrete nouns. We included four additional pairs. The task alternated between four learning blocks and four memory blocks. During the learning blocks, participants heard the twelve word pairs spoken by a male voice through headphones (with a 3 second inter-trial interval). Here, participants were instructed to memorise the pairings. During the memory blocks, they were prompted with one of the word pairs and were required to type its associate from the learning blocks (prompts and associates were the same throughout). Corrective feedback was provided onscreen (duration of 1.5 seconds). Participants completed the delayed block without an additional learning block, and no feedback was provided during this block. Presentation order of pairings was pseudorandomised between participants.

The fractal recognition stimuli were similar to those used in a previous study [40]. Sixty greyscale fractal images were used in total. Stimulus size was 400 x 400 pixels. The images were split into two sets of thirty. One set was used as target stimuli and the other set as distractors (counterbalanced so each list was used as targets or distractors for 50% of the participants). Participants were shown the target images during an encoding phase. Each image was presented for 1 second. Participants were instructed to press N on the keyboard as soon as the stimulus disappeared, to maintain attention. A new stimulus was presented upon key press. During the test phase, target and distractor images were randomly intermixed between participants. Participants had to indicate whether each image was ‘old’ or ‘new’ via a button press on the keyboard (Z and M, respectively). At test, images were presented for one second, and the next image was presented after the participant’s response.

Participants completed the associative learning tasks first (half completed visual first, half completed verbal first) before the fractal recognition task. After the retention interval (during which they performed visual psychophysics tasks) they completed the delayed blocks of the associative learning tasks in the same order as the learning blocks. Finally they completed the full, original 84 item SCSQ (note they only completed the questionnaire once).

### Results and Discussion

Data are included in S2 File. Firstly, we consider performance on the memory tasks before investigating the relationships between memory task performance and scores on the SCSQ subscales (note that we do not consider gender in the following analyses due to the small number of males in the sample). The proportions of correct responses for the verbal and visual paired associates tasks were calculated (see Table 4 for descriptive statistics). Considering performance on the visual paired associates task, there was a significant linear trend over the learning blocks, *F*(1, 43) = 66.78, *p* < .001, η^2^_*p*_ = .61, indicating performance improved over the blocks, and performance in each block differed significantly from performance in every other block, *ps* ≤ .001. Considering performance on the verbal paired associates task, there was a significant linear trend over the learning blocks, *F*(1, 43) = 80.15, *p* < .001, η^2^_*p*_ = .65, indicating performance improved over the blocks, and performance in each block differed significantly from performance in every other block, *ps* ≤ .002. Thus we can be confident that learning occurred during both the visual and verbal paired associates tasks.

**Table 4:** Mean proportion of correct responses over blocks for the visual and verbal paired associates tasks.

Task	Block 1	Block 2	Block 3	Block 4	Average	Delayed
Visual associates	.66 (.04)	.86 (.03)	.93 (.02)	-	.82 (.02)	.95 (.01)
Verbal associates	.60 (.05)	.88 (.03)	.94 (.02)	.97 (.01)	.85 (.02)	.97 (.01)

*Note*. Average refers to mean performance over blocks 1–3 for visual associates and blocks 1–4 for verbal associates. Standard errors in parentheses.

Performance in the fractal recognition task was measured via the signal detection measure, d’. This statistic represents the difference between standardised hit rate (correctly endorsing test stimuli present during learning) and false alarm rate (erroneously endorsing test stimuli not present during learning). Above chance recognition is shown if d’ is greater than zero. Mean d’ was 0.92 (*SE* = .09), which was significantly greater than zero, *t*(43) = 10.33, *p* < .001. Thus sufficient discrimination between targets and lures was demonstrated.

We then correlated task performance, averaging across the initial learning blocks in the paired associates tasks, with scores on the questionnaire subscales (see Table 5). *Technical/Spatial* scores correlated significantly with average visual associates performance, visual associates delayed performance, and fractal recognition d’. *Language & Word Forms* correlated significantly with average verbal associates performance and verbal associates delayed performance. Thus these factors demonstrated convergent validity. Perhaps surprisingly, *Imagery Ability* did not correlate significantly with performance on these tasks. In the visual associates task, participants were required to associate abstract line drawings with colours and in the fractal recognition task were required to recognise abstract images, thus performance on these tasks may be plausibly related to more abstract representational styles (captured by *Technical/Spatial* scores), rather than concrete representational style (captured by *Imagery Ability* scores). Convergent validity of the *Imagery Ability* factor could potentially be demonstrated using more naturalistic stimuli (such as natural scenes). *Global Bias* correlated negatively with performance in the learning blocks of the Visual Paired Associates task, indicating better performance was related to local bias. However, this factor did not correlate with performance in the delayed block. This could indicate that local bias confers a benefit to memory encoding when associating complex, abstract shapes with other visual materials. It may also reflect a Type I error (given the number of analyses performed we would predict 1.5 significant results by chance). Although most results would not survive a Bonferroni correction for multiple comparisons, the significant results represent medium effect sizes [37], generally form a stable pattern across measures, and are in line with theory.

**Table 5:** Correlation coefficients (Spearman’s rho) between SCSQ factor scores and memory task performance.

Task	Imagery Ability	Technical / Spatial	Language & Word Forms	Need for Organisation	Global Bias	Systemising Tendency
Visual associates—learning blocks	.26	.31*	.16	.25	-.31*	.15
Visual associates—delayed recall	-.07	.33*	-.13	.23	.12	-.20
Fractal recognition d’	.13	.31*	.07	-.04	.03	-.05
Verbal associates—learning blocks	.28	.10	.40**	-.13	-.22	.21
Verbal associates—delayed recall	.00	.02	.32*	.12	-.17	.06

*Note*. ‘Visual associates—learning’ refers to mean performance over blocks 1–3 on this task. ‘Verbal associates—learning’ refers to mean performance on blocks 1–4 on this task.

* *p* < .05;

** *p* < .01.

## Study 3

In Study 3 we sought to validate the SCSQ subscales identified in Study 1 by issuing it to a sample of participants with atypical experiences, namely grapheme-colour and sequence-space synaesthetes. It has been suggested that synaesthetes’ cognitive preferences may reflect affinities for materials associated with their synaesthesia, either in the domain that induces the experience, or the domain of the experience itself [10]. Thus, in the case of grapheme-colour synaesthesia we would expect a higher rating than that of non-synaesthetes on verbal and visual styles (here, *Language & Word Forms* and *Imagery Ability* respectively) because the inducer is verbal and the concurrent is visual.

Although the bulk of research on imagery, cognitive styles and individual differences in synaesthesia has focused on grapheme-colour variants (e.g., [10,31,41]), recent work has begun to address imagery in sequence-space synaesthesia. For example, since sequence-space synaesthetes appear to be superior in tasks of mental rotation (e.g., [42,43]; but see [44]) we might predict they would score highly in our questionnaire on spatial imagery. Interestingly, these synaesthetes had not scored highly on previous questionnaires of spatial imagery [45], which conflicts with their performance in the equivalent behavioural task (e.g., rotation; [42]). It may therefore be possible that previous self-report questionnaires were failing to detect spatial skills in this group. This may be because spatial imagery forms part of constellation of abilities, as shown by the *Technical/Spatial* factor, and only by taking into account related abilities would we observe the posited advantage. Additionally, as spatial abilities and systemising are correlated [15], we sought to determine whether sequence-space synaesthetes in particular would also score highly on *Systemising Tendency*. We therefore look with interest at how sequence-space synaesthetes might perform in our own questionnaire, particularly in relation to spatial imagery and systemising. Such results would help verify these subscales of the SCSQ.

### Method

#### Participants

We tested 121 synaesthete participants, 78 with sequence-space synaesthesia, 22 with grapheme-colour synaesthesia and 21 with both. Participants were recruited by email from a database of synaesthete volunteers at the University of Sussex (these were not the same self-declared synaesthetes excluded from the analysis in Study 1). The consent procedure was the same as used as in Study 1.

All individuals completed a questionnaire detailing their synaesthetic experiences, and participants were classified as having sequence-space experiences on the basis of answering affirmatively to the question “Some people always experience sequences in a particular spatial arrangement. Do you think this applies to you?”. Example diagrams of spatial forms were given when answering this question. All indicated having sequence-space synaesthesia for at least one of the following sequences: Numbers, days, months, years, letters of the alphabet, temperature, height, weight. Grapheme-colour synaesthetes were first identified from a question asking whether they experience coloured letters or digit, but were further validated using the most widely available online testing tool hosted at www.synesthete.org (for detailed methods see [46]). In this test, participants are shown each grapheme (letters A-Z, digits 0–9) three times in a random order and must select their synaesthetic colours from an online palette of colours. Synaesthetes typically show very high internal consistency in this test (e.g., repeatedly giving the same shade of red for the letter A) and this metric is used to generate a standardised colour-distance score. A score below 1.43 indicates the consistency typical of grapheme-colour synaesthetes (e.g., [47,48]). Our 43 grapheme-colour synaesthetes all scored ≤ 1.43 (*M* = 0.76, *SD* = 0.25).

The mean age of our synaesthete participants was 35 years (*SD* = 15) and 80% were female. To match the sample in terms of gender, we took a stratified random sample from the control group (Study 1 participants) to yield a gender ratio of 80:20 female:male participants. To maximise use of the data, nine gender-matched controls were randomly selected for each synaesthete (874 females, 216 males).

#### Materials and procedure

Synaesthetes completed the SCSQ via Bristol Online Surveys in the same manner reported for Study 1. Mean factor scores were calculated for each participant for each subscale of the SCSQ.

### Results and Discussion

Data are included in S3 File. The results are summarised in Table 6. The three groups of synaesthete are defined by the presence of either or both types of synaesthesia and the controls are defined by the absence of both.

**Table 6:** Mean factor scores as a function of presence/absence of grapheme-colour and sequence-space synaesthesia.

Grapheme-Colour	Sequence-Space	Imagery Ability	Technical / Spatial	Language & Word Forms	Need for Organisation	Global Bias	Systemising Tendency
Present	Present	4.22 (.09)	3.06 (.18)	4.34 (.15)	3.08 (.15)	2.74 (.12)	2.85 (.18)
	Absent	3.95 (.11)	3.02 (.13)	4.23 (.12)	3.03 (.20)	2.72 (.14)	2.96 (.16)
Absent	Present	4.16 (.06)	3.06 (.07)	4.06 (.08)	3.18 (.09)	2.53 (.08)	3.25 (.09)
	Absent	3.67 (.02)	2.83 (.02)	3.52 (.02)	3.11 (.02)	2.98 (.02)	2.62 (.02)

*Note*. Standard error appear in parentheses.

We conducted separate multiple regression analyses to determine whether the presence of different types of synaesthesia predicted scores on each of the SCSQ subscales. It is important to note that individuals with synaesthesia often have multiple forms (e.g., [49]). By specifically controlling for this in the regression analyses, we can address which forms of synaesthesia contribute most to different forms of cognitive styles. The presence/absence of grapheme-colour and sequence-space synaesthesia were coded as 0 (absent) and 1 (present) and entered into regression models as binary predictors for each SCSQ subscale. Importantly, Study 1 revealed gender differences on the *Imagery Ability*, *Technical/Spatial*, and *Systemising Tendency* subscales. Therefore we entered gender as a third predictor into each regression to assess whether effects of synaesthesia were detectible when accounting for gender differences. Females were coded as 1 and males as 0.

Table 7 shows the results of the regression analyses. Both grapheme-colour synaesthesia and sequence-space were linked to increased *Imagery Ability* and increased *Language and Word forms* scores. Sequence-space synaesthesia, but not grapheme-colour synaesthesia, was linked to increased *Technical/Spatial*, increased *Systemising Tendency* and reduced *Global Bias* (i.e. a greater local bias) scores. The differences observed between grapheme-colour and sequence-space synaesthetes on SCSQ scales shows that different forms of synaesthesia may predict different aspects of cognition, and should be accounted for in future research.

**Table 7:** Regression weights (unstandardised *b*, standard error, and standardised β) for each subscale of the SCSQ, as a function of the presence/absence of grapheme-colour synaesthesia, sequence-space synaesthesia, and gender.

Factor	Predictor	*B*	SE	*Β*	*t*
Imagery Ability	Grapheme-colour	.18	.08	.06	2.18*
	Sequence-Space	.46	.06	.24	8.18***
	Gender	.11	.04	.08	2.83**
Technical/Spatial	Grapheme-colour	.14	.09	.04	1.55
	Sequence-Space	.19	.06	.08	2.97**
	Gender	-.66	.04	-.41	15.87***
Language & Word Forms	Grapheme-colour	.52	.12	.13	4.39***
	Sequence-Space	.49	.08	.18	6.11***
	Gender	.09	.05	.05	1.74
Need for Organisation	Grapheme-colour	-.08	.12	-.02	0.69
	Sequence-Space	.07	.08	.03	0.82
	Gender	-.06	.05	-.03	1.15
Global bias	Grapheme-colour	-.05	.10	-.02	0.52
	Sequence-Space	-.38	.07	-.18	5.92***
	Gender	-.05	.04	-.03	1.18
Systemising Tendency	Grapheme-colour	.04	.11	.01	0.34
	Sequence-Space	0.52	.08	.20	6.72***
	Gender	-.21	.05	-.12	4.16***

Note:

* p < .05,

** p < .01,

*** p < .001.

Gender is coded as 1 = female and 0 = male. Positive scores indicate females scored higher on this factor. Negative scores indicate males scored higher.

The relationship between gender and SCSQ scores reflects that observed in Study 1, even when accounting for synaesthesia. Females scored higher on *Imagery Ability*, and males scored higher on both *Technical/Spatial*, and *Systemising Tendency*. Thus gender and the presence of different forms of synaesthesia make independent contributions to SCSQ scores.

## General Discussion

The current research aimed to produce a new cognitive styles questionnaire—the SCSQ—that links previously unconnected domains: the verbaliser-visualiser tradition alongside global/local processing and systemising. To achieve this, we issued the SCSQ to a large number of participants and assessed its factor structure and reliability. We found six factors: *Imagery Ability*, *Technical/Spatial*, *Language & Word Forms*, *Need for Organisation*, *Global Bias*, and *Systemising Tendency* which all exhibited good levels of internal consistency. We also found generally low to modest, but statistically significant, correlations between almost all of the factors.

We replicated the finding that visual and verbal styles are separable [8,9] and that visual imagery is not a unitary construct, but can be subdivided further [11,13]. Here we found *Imagery Ability* and *Technical/Spatial* factors, when new items were included in the measure. We also found that global/local bias items formed their own factor, as did systemising items. Notably, each factor contained items from more than one source (i.e. the factor analysis did not simply cluster items according to their original measure). Furthermore, all factors (with the exception of *Need for Organisation*) demonstrated construct validity. Firstly Study 1 revealed gender differences on SCSQ subscales. Secondly SCSQ scores predicted performance on visual and verbal memory tasks in Study 2, in-line with previous findings that these cognitive styles predict objective task performance [5,6,7]. Thirdly, SCSQ scores differed according to the presence and absence of synaesthesia in Study 3.

The *Imagery Ability* factor is conceptually similar to the object visual factor from the OSIVQ containing a number of items related to detail in object imagery. Indeed, this factor contained six items from this subscale (and one negatively loading item from the spatial subscale of the OSIVQ). The *Imagery Ability* factor also contained items from a number of other sources—four items from the IDQ and six new items designed to capture the use of imagery in everyday life (e.g., “When I can’t find something I’m looking for, I automatically visualise the last place I saw it”). Thus it may be the case that self-rated object imagery is tightly linked with its use in everyday life.

Our *Technical/Spatial* factor focuses on spatial imagery and other activities based on abstract cognitive skills such as mathematical abilities and technical interests. This subscale contained seven items from the spatial subscale of the OSIVQ, but also contained six items from the SQ and two from the AQ, thus this latent factor captures more than spatial mental imagery. That mathematical items clustered with spatial imagery items perhaps is not surprising; mathematical problem solving has previously been shown to correlate positively with spatial imagery and negatively with pictorial imagery (e.g., [50]; see [51], for review) and latent factors driving performance on spatial imagery tasks could covary with mathematical ability (see [51] for the neuro-anatomical basis of numerical and spatial processes). Interestingly, previous factor analyses of the SQ show that items relating to interest in technology form their own component of systemising [15,52]. That items relating to an interest or concern with technical details also loaded on the *Technical/Spatial* factor also indicates a more general capacity for dealing with abstract information.

In terms of scores for our imagery factors, females rated themselves higher on *Imagery Ability* than males, and males rated themselves higher on *Technical/Spatial* than females. The findings with respect to gender and different forms of imagery are in good agreement with other studies [11,13,38], and thus validate the imagery subscales of the SCSQ.

Both the presence of grapheme-colour and sequence-space synaesthesia significantly predicted scores on the *Imagery Ability* factor. This supports previous research showing these groups rate themselves highly on object imagery (e.g., [10,42,43]). Additionally, sequence-space synaesthesia predicted scores on the *Technical/Spatial* factor where previous research failed to find an advantage for this group in terms of self-report spatial imagery [44,45], despite others’ sometimes showing greater objective spatial imagery skills (e.g., [42]). Our finding of superior scores in the *Technical/Spatial* factor is more in-line with most behavioural findings in this domain. Additionally, this may reflect the factor’s capture of abilities correlated with spatial processing, such as mathematics, on which synaesthetes may also differ. For example, 77% of a sample of sequence-space synaesthetes reported consciously using their number forms in calculations [53].

*Language & Word Forms* contained one item (pertaining to language) from the SQ and five new items. Most of the items on this subscale relate to the appearance of words and sentences (such as spelling and grammar structure), thus this factor emphasises orthographic qualities. That all language items formed their own factor supports the tripartite model of visual imagery and verbal skills (e.g., [13]), even though most of the verbal items used in the SCSQ we used were novel and de-emphasised oral aspects of verbal skills. In addition to *Imagery Ability* scores, the presence of grapheme-colour synaesthesia predicted scores on *Language & Word Forms*. This supports the hypothesis that synaesthetes’ cognitive styles relate to inducing- or concurrent-related information, i.e. verbal and visual information in the case of grapheme-colour synaesthesia, replicating findings by Meier and Rothen [10] who issued the VVQ to grapheme-colour synaesthetes. That the SCSQ performed similarly to the VVQ offers further validation. Interestingly, the presence of sequence-space synaesthesia also predicted scores on *Language & Word Forms*. Notably, letters of the alphabet are often cited as inducing spatial forms in sequence-space synaesthetes. This also fits well with Meier and Rothen’s [10] suggestion that synaesthetes’ cognitive styles reflect a particular affinity for their inducing materials.

*Need for Organisation* emerged as a factor, which contained one item from the SQ and five new items, and was not predicted a priori. Neither gender, nor the presence of either form of considered synaesthesia predicted scores on this factor. Future validation work could explore this factor’s relationship to orderliness factors on other questionnaire measures (e.g. Comrey Personality Scales, [54]).

*Global bias* contained three items from the AQ, four new items and one item from the OSIVQ object factor (which focused on attention to specific details). As globally oriented items loaded positively on this factor and locally oriented items negatively, it appears that self-reported global/local bias can be conceptualised as opposite ends of a single dimension. *Systemising Tendency* contained four items from the SQ, one item from the AQ and one new item. All items related to systemising, and in particular precision in categorisation (of dates, animals, plants etc.).

Although *Systemising Tendency* and *Global Bias* emerged as separate factors, there was a modest negative correlation between them which indicates that those who exhibit higher systemising are also likely to exhibit a local bias (cf. [21]). The correlation between *Global Bias* and *Systemising Tendency* supported the notion that these traits are related, but not identical, presumably as one needs to pay sufficient attention to detail to systemise effectively [17]. Males also scored more highly than females on *Systemising Tendency*, replicating work by others [19,15,22].

Bouvet et al. [28] and Dale and Arnell [29] suggest there may be a cognitive style that underpins performance on global/local tasks and this is the first attempt to create a self-report subscale that attempts to gauge this style. Future work should aim to further validate the *Global Bias* scale by assessing its relationship with objective tests of local and global processing, such as the Navon [23] task. It could be the case that scores on this subscale predict performance on this task, or predict the extent to which individuals are affected by inappropriate processing orientations in a more direct manner than the SQ-R [52]. This subscale may also provide a useful proxy for global/local orientations when laboratory-based testing is impractical (e.g. online data collection).

In terms of the relationship between processing orientation and imagery, we observed that increased systemising and local processing were associated with increased *Imagery Ability* and *Technical/Spatial* scores. Kozhevnikov et al. [12] suggest that spatial imagers encode and process images in an analytic manner to arrange and analyse components. Additionally, Kozhevnikov, Hegarty and Mayer [55] show that, although object visualisers tend to encode images as a single unit (global processing), they can also apply sequential, local reasoning in the verbal domains. This suggests that they may be adept at switching between processing orientations but self-report a local bias. The only non-significant correlation was between *Technical/Spatial* and *Language & Word Forms*.

The presence of sequence-space synaesthesia, but not grapheme-colour synaesthesia was associated with a lower *Global Bias* score (i.e. a local bias) and a higher *Systemising Tendency* score. Synaesthetic experiences are by their nature systematic (e.g., consistency between inducer and concurrent or application of rules to the spatial form to arrive at some outcome), and a proclivity to understand or construct systems is a hallmark of systemising. Being able to locally inspect, manipulate and analyse forms may well depend on local processing. For instance, Simner et al. [36] (also [56]) showed that synaesthetes with time-space forms have superior recall of dates and events in time and that most reported that target events were retrieved from their spatial forms. (See also [57], for a detailed phenomenological report of sequence-space synaesthesia.). Additionally, sequence-space synaesthesia is often linked to, amongst others, calendar and numerical systems, that themselves are often subject to systemising [20].

Finally, past research into cognitive styles has had trouble connecting theory with measurement [2,13]. We consolidated research from seemingly disparate areas (object, spatial and verbal processing, systemising, global/local bias, and synaesthesia) into a novel cognitive style measure that can be applied to many domains of research (e.g. in typical and atypical populations). In Study 3 we predicted how the subscales of the SCSQ would behave with respect to a special population of synaesthetes, and these predictions were largely borne out through validation with external measures.

## Supporting Information

S1 File Study 1 Data—Item scores on each SCSQ item. Item numbers are given, and can be crossed-referenced with S1 Table for the item wordings. Gender is coded as 0 = female; 1 = male.
(XLSX)

S2 File Study 2 Data—Factor scores and memory task performance. Mean factor scores for every participant have been calculated, and performance for each task given as a proportion of correct responses. Full data for each block in the associates tasks are included, along with d’ scores and response criterion for the fractal recognition task. Gender is coded as 0 = female; 1 = male.
(XLSX)

S3 File Study 3 Data—Factor scores and presence of synaesthesia. Mean factor scores for participants included in Study 3. Grapheme-colour synaesthesia is coded as 0 = absent; 1 = present. Sequence-space synaesthesia is coded as 0 = absent; 1 = present. Gender is coded as 0 = female; 1 = male. GCS score refers to the consistency scores for the Eagleman et al. [47] consistency test previously taken by grapheme-colour synaesthetes in Study 3.
(XLSX)

S1 Table Items used in the SCSQ, their original source (and subscale, if appropriate, in parentheses), and item number in the current measure. (DOCX)

S2 Table Tables A-F: Inter-item correlation matrices for each subscale of the SCSQ. (DOCX)

## References

[1] Messick S (1984). The nature of cognitive styles: Problems and promise in educational practice. Educ Psychol 19: 59–74. 10.1080/00461528409529283

[2] Kozhevnikov M (2007). Cognitive styles in the context of modern psychology: Toward an integrated framework of cognitive style. Psychol Bull 133: 464–481. 10.1037/0033-2909.133.3.464 17469987

[3] Paivio A (1971). Imagery and verbal processes. New York: Holt, Rinehart and Winston.

[4] Richardson A (1977). Verbalizer-Visualizer: A cognitive style dimension. Journal of Mental Imagery, 1, 109–126.

[5] Kraemer DJM, Rosenberg LM, Thompson-Schill SL (2009). The neural correlates of visual and verbal cognitive styles. J Neurosci 29: 3792–3798. 10.1523/JNEUROSCI.4635-08.2009 10.1523/JNEUROSCI.4635-08.2009 19321775 PMC2697032

[6] Hsu NNS, Kraemer DJM, Oliver RT, Schlicting ML, Thompson-Schill SL (2011). Colour, context, and cognitive style: Variations in colour knowledge retrieval as a function of task and subject variables. J Cogn Neurosci 23: 2544–2557. 10.1162/jocn.2011.21619 10.1162/jocn.2011.21619 21265605

[7] Miller MB, Donovan C, Bennett CM, Aminoff EM, Mayer RE (2012). Individual differences in cognitive style and strategy predict similarities in the patterns of brain activity between individuals. Neuroimage 59: 83–93. 10.1016/j.neuroimage.2011.05.060 10.1016/j.neuroimage.2011.05.060 21651986

[8] Kirby JR, Moore PJ, Schofield NJ (1988). Verbal and visual learning. Contemp Educ Psychol 13: 169–184. 10.1016/0361-476X(88)90017-3

[9] Paivio A, Harshman R (1983). Factor analysis of a questionnaire on imagery and verbal habits and skills. Can J Exp Psychol 37: 461–483. 10.1037/h0080749

[10] Meier B, Rothen N (2013). Grapheme-colour synaesthesia is associated with a distinct cognitive style. Front Psychol 4 10.3389/fpsyg.2013.00632 PMC3777024 24065938

[11] Blajenkova O, Kozhevnikov M, Motes MA (2006). Object-spatial imagery: A new self-report imagery questionnaire. Appl Cogn Psychol 20: 239–263. 10.1002/acp.1182

[12] Kozhevnikov M, Kosslyn S, Shephard J (2005). Spatial versus object visualisers: A new characterisation of visual cognitive style. Mem Cognit 33: 710–726. 10.3758/BF03195337 16248335

[13] Blazhenvoka O, Kozhevnikov M (2009). The new object-spatial-verbal cognitive style model: Theory and measurement. Appl Cogn Psychol 23: 638–663. 10.1002/acp.1473

[14] Brosnan M, Dagga R, Collomose J (2010). The relationship between systemising and mental rotation and the implications for the extreme male brain theory of autism. J Autism Dev Disord 40: 1–7. 10.1007/s10803-009-0815-3 10.1007/s10803-009-0815-3 19633942

[15] Ling J, Burton TC, Salt JL, Muncer SJ (2009). Psychometric analysis of the systemising quotient (SQ) scale. Br J Psychol 100: 539–52. 10.1348/000712608X368261 10.1348/000712608X368261 19026108

[16] Morsanyi K, Primi C, Handley SJ, Chiesi F, Galli S (2012). Are systemising and autistic traits related to talent and interest in mathematics and engineering? Testing some of the central claims of the empathising-systemising theory. Br J Psychol 103: 472–496. 10.1111/j.2044-8295.2011.02089.x 10.1111/j.2044-8295.2011.02089.x 23034108

[17] Baron-Cohen S (2008). Autism, hypersystemising, and truth. Q J Exp Psychol 61: 64–75. 10.1080/17470210701508749 18038339

[18] Baron-Cohen S, Ashwin E, Ashwin C, Tavassoli T, Chakrabarti B (2009). Talent in autism: Hyper-systemising, hyper-attention to detail and sensory hypersensitivity. Philos Trans R Soc Lond B Biol Sci 364: 1377–1383. 10.1098/rstb.2008.0337 10.1098/rstb.2008.0337 19528020 PMC2677592

[19] Baron-Cohen S, Richler J, Bisarya D, Gurunathan N, Wheelwright S (2003). The systemising quotient: An investigation of adults with Asperger syndrome or high-functioning autism, and normal sex differences. Philos Trans R Soc Lond B Biol Sci 358: 361–374. 10.1098/rstb.2002.1206 12639333 PMC1693117

[20] Baron-Cohen S (2009). Autism: The empathising-systemising (E-S) theory. Ann N Y Acad Sci 1156, 68–80. 10.1111/j.1749-6632.2009.04467.x 19338503

[21] Billington J, Baron-Cohen S, Bor D (2008). Systemising influences attentional processes during the Navon task: An fMRI study. Neuropsychologia 46: 511–520. 10.1016/j.neuropsychologia.2007.09.003 17963797

[22] Wheelwright S, Baron-Cohen S, Goldenfeld N, Delaney J, Fine D, Smith R et al (2006). Predicting Autism Spectrum Quotient (AQ) from the Systemising Quotient-Revised (SQ-R) and Empathy Questionnaire (EQ). Brain Res 1079: 47–56. 10.1016/j.brainres.2006.01.012 16473340

[23] Navon D (1977). Forest before trees: The precedence of global features in visual perception. Cogn Psychol 9: 363–383. 10.1016/0010-0285(77)90012-3

[24] Basso MR, Lowery N (2004). Global-local visual biases correspond with visual-spatial orientation. J Clin Exp Neuropsychol 26: 24–30. 10.1076/jcen.26.1.24.23939 14972691

[25] Macrae CN, Lewis HL (2002). Do I know you? Processing orientation and face recognition. Psychol Sci 13: 194–196. 10.1111/1467-9280.00436 11934008

[26] Brown C, Lloyd-Jones TJ (2002). Verbal overshadowing in a multiple face presentation paradigm: Effects of description instruction. Appl Cogn Psychol 16: 873–885. 10.1002/acp.919

[27] Brown C, Lloyd-Jones TJ (2003). Verbal overshadowing of multiple face and car recognition: Effects of within- versus across-category verbal descriptions. Appl Cogn Psychol 17: 183–201. 10.1002/acp.861

[28] Bouvet L, Rousset S, Valdois S, Donnadieu S (2011). Global precedence effect in audition and vision: Evidence for similar cognitive styles across modalities. Acta Psychol 138: 239–335. 10.1016/j.actpsy.2011.08.004 21943833

[29] Dale G, Arnell KM (2013). Investigating the stability of and relationships among global/local processing measures. Atten Percept & Psychophys 75: 394–406. 10.3758/s13414-012-0416-7 23354593

[30] Barnett KJ, Newell FN (2008). Synaesthesia is associated with enhanced, self-rated visual imagery. Conscious Cogn 17: 1032–1039. 10.1016/j.concog.2007.05.011 17627844

[31] Grossenbacher PG, Lovelace CT (2001). Mechanisms of synaesthesia: Cognitive and physiological constraints. Trends Cogn Sci 5: 36–41. 10.1016/S1364-6613(00)01571-0 11164734

[32] Simner J (2007). Beyond perception: Synaesthesia as a psycholinguistic phenomenon. Trends Cogn Sci 11: 23–29. 10.1016/j.tics.2006.10.010 17137829

[33] Simner J (2009). Synaesthetic visuo-spatial forms: Viewing sequences in space. Cortex, 45, 1138–1147. 10.1016/j.cortex.2009.07.001 10.1016/j.cortex.2009.07.001 19664765

[34] Baron-Cohen S, Wheelwright S, Skinner R, Martin J, Clubley E (2001). The autism-spectrum quotient (AQ): Evidence from Asperger syndrome/high-functioning autism, males and females, scientists and mathematicians. J Autism Dev Disord 31: 5–17. 10.1023/A:1005653411471 11439754

[35] Hiscock M (1978). Imagery assessment through self-report: What do imagery questionnaires measure? J Consult Clin Psychol 46: 223–230. 10.1037/0022-006X.46.2.223 649798

[36] Velicer WF (1976). Determining the number of components from the matrix of partial correlations. Psychometrika 41: 321–327. 10.1007/BF02293557

[37] Cohen J (1992). A power primer. Psychol Bull 112: 115–159. 10.1037/0033-2909.112.1.155 19565683

[38] Campos A (2014). Gender differences in imagery. Pers Indiv Differ 59: 107–111 10.1016/j.paid.2013.12.010

[39] Wechsler DA (1987). Wechsler Memory Scale-Revised Manual. New York: Psychological Corporation

[40] Ward J, Hovard P, Jones A, Rothen N (2013). Enhanced recognition memory in grapheme-colour synaesthesia for different categories of visual stimuli. Front Psychol 4 10.3389/fpsyg.2013.00762 PMC3807560 24187542

[41] Spiller MJ, Jansari AS (2008). Mental imagery and synaesthesia: Is synaesthesia from internally generated stimuli possible? Cognition 109: 143–151. 10.1016/j.cognition.2008.08.007 10.1016/j.cognition.2008.08.007 18834583

[42] Simner J, Mayo J, Spiller MJ (2009). A foundation for savantism? Visuo-spatial synaesthetes present with cognitive benefits. Cortex 45: 1246–1260. 10.1016/j.cortex.2009.07.007 10.1016/j.cortex.2009.07.007 19665699

[43] Brang D, Miller LE, McQuire M, Ramachandran VS, Coulson S (2013). Enhanced mental rotation in time-space synaesthesia. Cogn Process 14: 429–434. 10.1007/s10339-013-0561-5 10.1007/s10339-013-0561-5 23553317

[44] Rizza A, Price MC (2012). Do sequence-space synaesthetes have better spatial imagery skills? Maybe not. Cogn Process 13: 299–303. 10.1007/s10339-012-0459-7 22802032

[45] Price MC (2009). Spatial forms and mental imagery. Cortex 45: 1229–1245. 10.1016/j.cortex.2009.06.013 10.1016/j.cortex.2009.06.013 19665116

[46] Eagleman DM, Kagan AD, Nelson SS, Sagaram D, Sarma AK (2007). A standardised test battery for the study of synaesthesia. J Neurosci Methods 159: 139–145. 10.1016/j.jneumeth.2006.07.012 16919755 PMC4118597

[47] Rothen N, Seth AK, Witzel C, Ward J (2013). Diagnosing synaesthesia with online colour pickers: Maximising sensitivity and specificity. J Neurosci Methods 215: 156–160. 10.1016/j.jneumeth.2013.02.009 10.1016/j.jneumeth.2013.02.009 23458658

[48] Carmichael DA, Down MP, Shillcock RC, Eagleman DM, Simner J (2014). Validating a standardised test battery for synaesthesia: Does the Synaesthesia Battery reliably detect synaesthesia? Conscious Cogn 33: 375–385. 10.1016/j.concog.2015.02.001 PMC5047354 25734257

[49] Simner J, Mulvenna C, Sagiv N, Tsakanikos E, Witherby SA, Fraser C et al (2006). Synaesthesia: The prevalence of atypical cross-modal experiences. Perception 35: 1024–1033. 10.1068/p5469 17076063

[50] Hegarty M, Kozhevnikov M (1999). Types of visual-spatial representations and mathematical problem solving. J Educ Psychol 91: 684–689. 10.1037/0022-0663.91.4.684

[51] de Hevia MD, Vallar G, Girelli L (2008). Visualising numbers in the mind's eye: The role of visuo-spatial processes in numerical abilities. Neurosci Biobehav Rev 32: 1361–1372. 10.1016/j.neubiorev.2008.05.015 10.1016/j.neubiorev.2008.05.015 18584868

[52] Wakabayashi A, Baron-Cohen S, Wheelwright S, Goldenfeld N, Delaney J, Fine D et al (2006). Development of short forms of the empathy quotient (EQ-short) and the systemising quotient (SQ-short). Pers Individ Dif 41: 929–940. 10.1016/j.paid.2006.03.017

[53] Ward J, Sagiv N, Butterworth B (2009). The impact of visuo-spatial number forms on simple arithmetic. Cortex 45: 1261–1265. 10.1016/j.cortex.2009.03.017 10.1016/j.cortex.2009.03.017 19631317

[54] Hahn R, Comrey AL (1994). Factor analysis of the NEO-PI and The Comrey Personality Scales. Psychol Rep 75: 355–365. 10.1016/0191-8869(94)00156-M

[55] Kozhevnikov M, Hegarty M, Mayer RE (2002). Revising the visualiser-verbaliser dimension: Evidence for two types of visualisers. Cogn Instr 20: 47–77. 10.1207/S1532690XCI2001_3

[56] Mann H, Korzenko J, Carriere JSA, Dixon MJ (2009). Time-space synaesthesia—A cognitive advantage? Conscious Cogn 18: 619–627. 10.1016/j.concog.2009.06.005 10.1016/j.concog.2009.06.005 19632133

[57] Gould C, Froese T, Barrett AB, Ward J, Seth AK (2014). An extended case study on the phenomenology of sequence-space synaesthesia. Front Hum Neurosci 8 10.3389/fnhum.2014.00433 PMC4080762 25071498

