# Supplementary material for: Different Dimensions of Cognitive Style in Typical and Atypical Cognition: New Evidence and a New Measurement Tool
Source: PLoS One. 2016 May 18;11(5):e0155483. doi: 10.1371/journal.pone.0155483 (PMC4871558; doi:10.1371/journal.pone.0155483)
Supplement: S1 Table — (DOCX) [file pone.0155483.s004.docx]

**S1 Table.** Items used in the SCSQ, their original source (and subscale, if appropriate, in parentheses), and item number in the current measure

| Item number | Original measure | Item |
| --- | --- | --- |
| 1 | New item | I enjoy learning languages |
| 2 | OSIVQ (Spatial Imagery) | I find it difficult to imagine how a three-dimensional geometric figure would exactly look like when rotated |
| 3 | New item | When listening to music, I listen to the whole piece rather than the contribution of individual instruments |
| 4 | SQ | I am interested in knowing the path a river takes from its source to the sea. |
| 5 | OSIVQ (Spatial Imagery) | My mental images are more schematic than colourful and pictorial |
| 6 | New item | When I'm planning to do a complex or difficult task, I visualise myself doing it first |
| 7 | SQ | When I read something, I always notice whether it is grammatically correct. |
| 8 | IDQ (Habitual use of Imagery) | I have only vague visual impressions of scenes I have experienced. |
| 9 | SQ | In maths, I am intrigued by the rules and patterns governing numbers. |
| 10 | IDQ (Habitual use of Imagery) | When remembering a scene, I use verbal descriptions rather than mental pictures. |
| 11 | New item | When I think of historical events, the exact date is important to me |
| 12 | OSIVQ (Object Imagery) | I enjoy pictures with bright colours and unusual shapes like the ones in modern art |
| 13 | SQ | When an election is being held, I am not interested in the results for each constituency. |
| 14 | SQ | When I listen to a piece of music, I always notice the way it’s structured. |
| 15 | OSIVQ (Object Imagery) | I can easily remember a great deal of visual details that someone else might never notice. For example, I would just automatically take some things in, like what colour is a shirt someone wears or what colour are his/her shoes |
| 16 | New item | I tend to focus on details in a scene rather than the whole picture |
| 17 | OSIVQ (Spatial Imagery) | I am good in playing spatial games involving constructing from blocks and paper (e.g. Lego, Tetris, Origami) |
| 18 | OSIVQ (Spatial Imagery) | In school, I had no problems with geometry |
| 19 | New item | When I picture the route somewhere, I visualise that route as if I were walking/driving/cycling it |
| 20 | New item | Order is important to me |
| 21 | New item | I like to group things together under a single label |
| 22 | IDQ (Habitual use of Imagery) | I often enjoy the use of mental pictures to reminisce. |
| 23 | OSIVQ (Object Imagery) | When I hear a radio announcer or a DJ I’ve never actually seen, I usually find myself picturing what he or she might look like |
| 24 | SQ | When I am walking in the country, I am curious about how the various kinds of trees differ. |
| 25 | AQ (Attention to Detail) | I don't usually notice small changes in a situation or a person's appearance |
| 26 | OSIVQ (Object Imagery) | If I were asked to choose among engineering professions, or visual arts, I would choose visual arts |
| 27 | New item | When I think of a face, I imagine it as a whole rather than focus on individual features |
| 28 | AQ (Attention to Detail) | I am fascinated by dates |
| 29 | SQ | When I look at an animal, I like to know the precise species it belongs to. |
| 30 | New item | Certain dates are significant to me without obvious reason (such as a holiday or friend's birthday) |
| 31 | SQ | I find it easy to grasp exactly how odds work in betting. |
| 32 | New item | When I hear a new word, I am curious to know how it is spelled |
| 33 | AQ (Attention to Detail) | I am fascinated by numbers |
| 34 | New item | I plan my diary using a mental image of time |
| 35 | SQ | I am not very meticulous when I carry out D.I.Y. |
| 36 | OSIVQ (Object Imagery) | My mental images are very vivid and photographic |
| 37 | AQ (Attention to Detail) | I am not very good at remembering phone numbers |
| 38 | New item | I keep my book collection organised alphabetically |
| 39 | OSIVQ (Object Imagery) | I have a photographic memory |
| 40 | OSIVQ (Spatial Imagery) | I can easily sketch a blueprint for a building I am familiar with |
| 41 | OSIVQ (Spatial Imagery) | I can easily imagine and mentally rotate three-dimensional geometric figures |
| 42 | OSIVQ (Spatial Imagery) | My graphic abilities would make a career in architecture relatively easy for me |
| 43 | OSIVQ (Object Imagery) | I can close my eyes and easily picture a scene that I have experienced |
| 44 | OSIVQ (Spatial Imagery) | I prefer schematic diagrams and sketches when reading a textbook instead of colourful and pictorial illustrations |
| 45 | New item | I find it easy to group things together under a single label |
| 46 | OSIVQ (Spatial Imagery) | When thinking about an abstract concept (or building), I imagine an abstract schematic building in my mind or its blueprint rather than a specific concrete building |
| 47 | New item | I like learning new words |
| 48 | SQ | I do not enjoy games that involve a high degree of strategy. |
| 49 | SQ | When I look at a piece of furniture, I do not notice the details of how it was constructed. |
| 50 | New item | I tend to think of numbers as arranged in space |
| 51 | New item | When I can't find something I'm looking for, I automatically visualise the last place I saw it |
| 52 | SQ | If I had a collection (e.g. CDs, coins, stamps), it would be highly organised. |
| 53 | New item | When I look at a tree I focus on its features such as branches and leaves rather than the whole |
| 54 | OSIVQ (Object Imagery) | I remember everything visually. I can recount what people wore to a dinner and I can talk about the way they sat and the way they looked probably in more detail than I could discuss what they said |
| 55 | SQ | When I read the newspaper, I am drawn to tables of information, such as football league scores or stock market indices. |
| 56 | New item | My memories are mainly visual in nature |
| 57 | AQ (Attention to Detail) | I notice patterns in things all the time |
| 58 | New item | I tend to omit small visual details in scenes I remember |
| 59 | SQ | I do not care to know the names of the plants I see. |
| 60 | OSIVQ (Object Imagery) | When reading fiction, I usually form a clear and detailed mental picture of a scene or room that has been described |
| 61 | SQ | If I were buying a stereo, I would want to know about its precise technical features. |
| 62 | IDQ (Habitual use of Imagery) | I often use mental images or pictures to help me remember things. |
| 63 | SQ | When I look at a building, I am curious about the precise way it was constructed. |
| 64 | New item | Mental imagery helps me remember things |
| 65 | New item | I tend to notice if a word has the same letter repeated in its spelling |
| 66 | SQ | I am fascinated by how machines work. |
| 67 | AQ (Attention to Detail) | I tend to notice details that others do not |
| 68 | SQ | If I were buying a camera, I would not look carefully into the quality of the lens. |
| 69 | OSIVQ (Spatial Imagery) | I have excellent abilities in technical graphics |
| 70 | SQ | When I buy a new appliance, I do not read the instruction manual very thoroughly. |
| 71 | New item | The spelling of words does not fascinate me |
| 72 | AQ (Attention to Detail) | I usually concentrate on the whole picture, rather than the small details |
| 73 | OSIVQ (Object Imagery) | When entering a familiar store to get a specific item, I can easily picture the exact location of the target item, the shelf it stands on, how it is arranged and the surrounding articles |
| 74 | OSIVQ (Spatial Imagery) | Architecture interests me more than painting |
| 75 | New item | When I picture the route somewhere, I visualise that route as if it were on a map |
| 76 | SQ | When I cook, I do not think about exactly how different methods and ingredients contribute to the final product. |
| 77 | New item | I keep my workspace highly organised (e.g. all files/folders on the same subject are in the same colour) |
| 78 | SQ | If I were buying a computer, I would want to know exact details about its hard drive capacity and processor speed. |
| 79 | New item | I get upset if my own methods of learning are impeded |
| 80 | SQ | When travelling by train, I often wonder exactly how the rail networks are coordinated. |
| 81 | OSIVQ (Object Imagery) | My mental images of different objects very much resemble the size, shape and colour of actual objects that I have seen |
| 82 | SQ | I find it difficult to understand instruction manuals for putting appliances together. |
| 83 | SQ | I can easily visualise how the motorways in my region link up. |
| 84 | New item | When I think of activities I have done, I do not remember in mental pictures |
