# Supplementary material for: Different Dimensions of Cognitive Style in Typical and Atypical Cognition: New Evidence and a New Measurement Tool
Source: PLoS One. 2016 May 18;11(5):e0155483. doi: 10.1371/journal.pone.0155483 (PMC4871558; doi:10.1371/journal.pone.0155483)
Supplement: S2 Table — (DOCX) [file pone.0155483.s005.docx]

**S2 Tables**

**Table A*.*** Inter-item Spearman correlations for Factor 1 (Imagery Ability)

| Item number | 62 | 64 | 56 | 22 | 36 | 84 | 43 | 60 | 81 | 10 | 51 | 8 | 19 | 73 | 5 | 6 |
| --- | --- | --- | --- | --- | --- | --- | --- | --- | --- | --- | --- | --- | --- | --- | --- | --- |
| 62 | - |  |  |  |  |  |  |  |  |  |  |  |  |  |  |  |
| 64 | .69 | - |  |  |  |  |  |  |  |  |  |  |  |  |  |  |
| 56 | .42 | .45 | - |  |  |  |  |  |  |  |  |  |  |  |  |  |
| 22 | .41 | .43 | .39 | - |  |  |  |  |  |  |  |  |  |  |  |  |
| 36 | .43 | .42 | .46 | .43 | - |  |  |  |  |  |  |  |  |  |  |  |
| 84 | -.40 | -.45 | -.41 | -.39 | -.39 | - |  |  |  |  |  |  |  |  |  |  |
| 43 | .41 | .40 | .40 | .41 | .49 | -.39 | - |  |  |  |  |  |  |  |  |  |
| 60 | .39 | .37 | .29 | .33 | .35 | -.32 | .34 | - |  |  |  |  |  |  |  |  |
| 81 | .32 | .30 | .32 | .27 | .29 | -.26 | .28 | .27 | - |  |  |  |  |  |  |  |
| 10 | -.25 | -.22 | -.29 | -.29 | -.25 | .35 | -.22 | -.19 | -.16 | - |  |  |  |  |  |  |
| 51 | .37 | .35 | .24 | .28 | .26 | -.26 | .29 | .28 | .23 | -.14 | - |  |  |  |  |  |
| 8 | -.26 | -.27 | -.25 | -.27 | -.38 | .31 | -.42 | -.21 | -.18 | .26 | -.16 | - |  |  |  |  |
| 19 | .29 | .26 | .21 | .26 | .21 | -.21 | .23 | .22 | .16 | -.13 | .27 | -.14 | - |  |  |  |
| 73 | .28 | .28 | .23 | .22 | .26 | -.19 | .28 | .23 | .28 | -.14 | .31 | -.18 | .23 | - |  |  |
| 5 | -.21 | -.22 | -.21 | -.19 | -.24 | .27 | -.16 | -.18 | -.10 | .17 | -.13 | .19 | -.07 | -.03 | - |  |
| 6 | .23 | .22 | .21 | .24 | .14 | -.17 | .20 | .18 | .21 | -.09 | .23 | -.06 | .24 | .19 | .04 | - |
| 23 | .23 | .20 | .14 | .22 | .20 | -.16 | .22 | .34 | .19 | -.10 | .19 | -.07 | .19 | .17 | -.10 | .21 |

**Table B.** Inter-item Spearman correlations for Factor 2 (Technical/Spatial)

| Item number | 41 | 18 | 2 | 9 | 42 | 33 | 17 | 31 | 69 | 40 | 44 | 61 | 78 | 55 | 57 | 48 |
| --- | --- | --- | --- | --- | --- | --- | --- | --- | --- | --- | --- | --- | --- | --- | --- | --- |
| 41 | - |  |  |  |  |  |  |  |  |  |  |  |  |  |  |  |
| 18 | .44 | - |  |  |  |  |  |  |  |  |  |  |  |  |  |  |
| 2 | -.72 | -.41 | - |  |  |  |  |  |  |  |  |  |  |  |  |  |
| 9 | .35 | .45 | -.28 | - |  |  |  |  |  |  |  |  |  |  |  |  |
| 42 | .53 | .32 | -.40 | .28 | - |  |  |  |  |  |  |  |  |  |  |  |
| 33 | .31 | .42 | -.24 | .65 | .26 | - |  |  |  |  |  |  |  |  |  |  |
| 17 | .44 | .41 | -.42 | .29 | .38 | .27 | - |  |  |  |  |  |  |  |  |  |
| 31 | .31 | .32 | -.20 | .36 | .23 | .43 | .24 | - |  |  |  |  |  |  |  |  |
| 69 | .38 | .27 | -.31 | .24 | .60 | .25 | .31 | .26 | - |  |  |  |  |  |  |  |
| 40 | .47 | .24 | -.32 | .23 | .57 | .23 | .31 | .21 | .40 | - |  |  |  |  |  |  |
| 44 | .28 | .27 | -.20 | .32 | .29 | .33 | .21 | .27 | .25 | .24 | - |  |  |  |  |  |
| 61 | .28 | .25 | -.23 | .26 | .22 | .27 | .20 | .30 | .29 | .23 | .28 | - |  |  |  |  |
| 78 | .30 | .22 | -.24 | .25 | .20 | .26 | .23 | .29 | .26 | .19 | .24 | .62 | - |  |  |  |
| 55 | .20 | .23 | -.14 | .26 | .25 | .35 | .16 | .39 | .25 | .24 | .31 | .28 | .20 | - |  |  |
| 57 | .31 | .22 | -.22 | .26 | .27 | .30 | .24 | .19 | .23 | .21 | .16 | .19 | .20 | .17 | - |  |
| 48 | -.19 | -.24 | .21 | -.21 | -.09 | -.22 | -.27 | -.27 | -.10 | -.11 | -.16 | -.15 | -.20 | -.18 | -.14 | - |
| 46 | .25 | .17 | -.15 | .25 | .34 | .26 | .17 | .18 | .33 | .33 | .31 | .20 | .13 | .23 | .13 | -.06 |

**Table C.** Inter-item Spearman correlations for Factor 3 (Language and Word Forms)

| Item number | 47 | 32 | 71 | 7 | 65 |
| --- | --- | --- | --- | --- | --- |
| 47 | - |  |  |  |  |
| 32 | .51 | - |  |  |  |
| 71 | -.47 | -.53 | - |  |  |
| 7 | .39 | .40 | -.36 | - |  |
| 65 | .30 | .37 | -.35 | .30 | - |
| 1 | .40 | .27 | -.27 | .18 | .14 |

**Table D.** Inter-item Spearman correlations for Factor 4 (Need for Organisation)

| Item number | 77 | 52 | 20 | 21 | 45 |
| --- | --- | --- | --- | --- | --- |
| 77 | - |  |  |  |  |
| 52 | .57 | - |  |  |  |
| 20 | .46 | .41 | - |  |  |
| 21 | .30 | .25 | .38 | - |  |
| 45 | .21 | .18 | .23 | .61 | - |
| 38 | .29 | .42 | .16 | .09 | .09 |

**Table E.** Inter-item Spearman correlations for Factor 5 (Global Bias)

| Item number | 72 | 16 | 15 | 67 | 25 | 58 | 27 |
| --- | --- | --- | --- | --- | --- | --- | --- |
| 72 | - |  |  |  |  |  |  |
| 16 | -.45 | - |  |  |  |  |  |
| 15 | -.28 | .44 | - |  |  |  |  |
| 67 | -.31 | .40 | .46 | - |  |  |  |
| 25 | .20 | -.21 | -.40 | -.28 | - |  |  |
| 58 | .24 | -.15 | -.23 | -.15 | .23 | - |  |
| 27 | .28 | -.12 | -.10 | -.07 | .11 | .10 | - |
| 53 | -.33 | .28 | .15 | .23 | -.09 | -.07 | -.19 |

**Table F.** Inter-item Spearman correlations for Factor 6 (Systemising Tendency)

| Item number | 24 | 59 | 4 | 29 | 28 |
| --- | --- | --- | --- | --- | --- |
| 24 | - |  |  |  |  |
| 59 | -.46 | - |  |  |  |
| 4 | .40 | -.30 | - |  |  |
| 29 | .35 | -.31 | .26 | - |  |
| 28 | .21 | -.12 | .22 | .31 | - |
| 11 | .18 | -.15 | .25 | .25 | .52 |
